# In Situ Construction of TiC-Ti_3_SiC_2_ Gradient Hybrid Interphase Coated SiC Fibers for Suppression of Specular Reflection and Non-Specular Scattering

**DOI:** 10.3390/ma16010292

**Published:** 2022-12-28

**Authors:** Haitang Yang, Yinrui Li, Heng Luo, Yangjun Zou, Jun He

**Affiliations:** 1 Chengdu Aircraft Industrial (Group) Co., Ltd., Chengdu 610092, China; 2 School of Physics and Electronics, Central South University, Changsha 410083, China; 3 State Key Laboratory of Powder Metallury, Central South University, Changsha 410083, China

**Keywords:** SiC fibers, Ti_3_SiC_2_, multilayer coating, molten salt, specular reflection, non-specular scattering

## Abstract

TiC-Ti_3_SiC_2_ gradient hybrid interphase on the surface of SiC fibers was successfully obtained through the molten salt method. The electromagnetic parameters of the prepared samples can be accurately controlled by adjusting the reaction temperature. A significant bimodal effect is observed in electromagnetic parameters patterns, corresponding to the double interface layer. TiC-Ti_3_SiC_2_ gradient hybrid interphase plays a dominant role in impedance matching, as well as in the attenuation layer through multi-interfacial polarization and conduction loss. Through the co-evaluation of the suppression of specular reflection and non-specular scattering properties of the samples, the SiC fiber with the TiC-Ti_3_SiC_2_ gradient hybrid interphase is expected to be a high temperature resistant radar absorbing material for future stealth aircraft.

## 1. Introduction

Through the combination of contour design and application of radar absorbing materials, the main scattering sources of an aircraft, namely cavity scattering, angular scattering and specular reflection, have been well suppressed [1,2,3,4,5,6]. However, with the development of radar detection technology, the deterioration of stealth performance caused by non-specular scattering is becoming more and more serious. The non-specular scattering mainly originates from surface wave propagation to electromagnetic discontinuities, such as stair, edge, gap, geometric and material mutation [7,8,9,10]. Therefore, for radar absorbing materials used in future stealth aircraft, its specular reflection and non-specular scattering suppression properties must be considered simultaneously.

With the advancement of material technology, the aircraft should have a larger Mach number of flight and a greater radar stealth performance in the future. The temperature of the skin and other components will be higher [11,12,13], which poses a great challenge to the application of radar absorbing materials. Therefore, radar absorbing materials in the future should have the characteristics of high-temperature resistance, high specific strength, oxidation resistance, creep resistance and great microwave absorbing properties. The SiC fiber-reinforced matrix composites with these characteristics will become the most likely candidate materials [14,15,16].

As one of the important components of SiC fiber-reinforced matrix composites, the interphase is the key to realizing the integration of structure bearing and microwave absorption. It is widely acknowledged that the interphase plays a dominant role in mechanical performance in terms of crack arresting or deflection. Meanwhile, it also acts as an electromagnetic impedance matching layer which determines the overall absorbing performance of composites. In spite of a number of microwave absorbing performances by single interphase coated SiC fibers that have been reported previously, such as PyC or BN interphase [17,18,19,20,21,22,23], the number of studies investigating the electromagnetic response of this unique gradient hybrid layer of modified SiC fibers with Ti_3_SiC_2_ interphase remain relatively few [24,25,26,27]. Therefore, it is quite essential to explore the microwave scattering and transmission behaviors of this bilayered coating on SiC fibers.

In this study, TiC-Ti_3_SiC_2_ gradient hybrid interphase coating on SiC fibers was first introduced through an in situ molten salt method to optimize the suppression of specular reflection and non-specular scattering. The microstructural and morphologies of the SiC fibers varying with Ti_3_SiC_2_ contents were characterized. The dielectric and microwave absorption properties of the fibers within 2–18 GHz were studied. Possible absorption mechanisms were proposed.

## 2. Experiment

### 2.1. Preparation of Ti_3_SiC_2_/SiC Fibers

Silicon carbide (SiC) fibers with a diameter of about 12 μm were provided from BoXiang Plant (China). Ti powders were used as a reactive metal source in this work. The molten salt mixture was composed of NaCl and KCl. Based on our previous work, the molar ratio of salt and titanium was fixed at NaCl:KCl:Ti = 4:4:1. Firstly, silicon carbide (SiC) fibers were placed in an alumina crucible and covered by a mixture of salt and titanium powders. The crucible was then put into a tube furnace and heated at evaluated temperatures (900 °C, 1000 °C, 1100 °C and 1200 °C) for 1 h under a flowing argon atmosphere. According to the heated temperature, the as-obtained samples were designated as S900, S1000, S1100 and S1200, respectively. For comparison, primary SiC fibers without any coating treatment were marked as S0. After cooling, the crucible was placed in distilled water to remove the salts and the coated SiC fiber samples were recovered after washing with distilled water and drying for 2 h at 60 °C in an oven.

### 2.2. Characterization

The phases compositions of the as-synthesized SiC fibers were identified by X-ray powder diffraction (XRD, XRD-7000, SHIMADZU, Kyoto, Japan) using Cu Kα radiation (λ = 0.154187 Å). The working voltage and current of the Cu target were 40 kV and 40 mA, respectively. A scanning electron microscope (SEM, Nova NanoSEM 450, FEI, Shanghai, China) equipped with an energy dispersive spectrometer (EDS) was used to examine the morphologies and to measure the thickness of the coating on fibers. The fiber samples were cut with scissors and exposed to a cross-section for scanning electron microscope observation. Then, the fibers were cut into 2 mm and mixed with paraffin at a fixed filler loading of 10 wt%, and then pressed into a cylindrical mold with a 7.00 mm outer diameter and 3.04 mm inner diameter. After that, samples were polished into a thickness of 2.5 mm. The complex permeability and permittivity of the test samples were examined over the frequency range of 2–18 GHz by an Agilent E5071C vector network analyzer through the reflection and transmission method.

## 3. Results and Discussion

### 3.1. Microstructure and Phase Analysis

**Figure 1:**
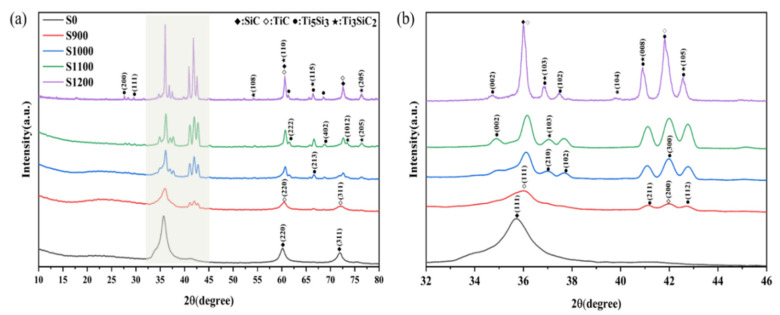
XRD (X-ray diffraction) patterns of SiC fiber tows coated with Ti_3_SiC_2_: (**a**) 10–80°; (**b**) 32–46°.

To investigate the composition evolution after sintering, XRD patterns of SiC fibers coated with different thicknesses of Ti_3_SiC_2_ sintered at 900~1200 °C are shown in Figure 1. As seen in Figure 1, the XRD pattern of the S0 sample shows three peaks at about 2θ = 35.73°, 60.14° and 71.97°, which are ascribed to the β-SiC(111), (220) and (311) crystal plane. When the sintering temperature was 900 °C, the XRD peaks of both TiC and Ti_5_Si_3_ phases could be observed. The diffraction peaks located at 2θ = 35.9°, 40.7°, 60.4° and 72.4° can be indexed to prove the formation of the TiC phase as the face-centered cubic structure (JCPDS No.32-1383). Peaks located at 2θ = 40.9° and 42.7° were ascribed to the (211) and (112) crystal planes of Ti_5_Si_3_. As the heat-treated temperature increases to 1000 °C, the intensity of diffraction peaks belonging to TiC increases, owing to the growth of the TiC crystals. In addition, three more peaks appeared at about 2θ = 37.05°, 37.65° and 66.78°, and corresponded to the (210), (102) and (213) crystal planes of Ti_5_Si_3_, respectively. As the heat-treated temperature increased to 1100 °C, three new peaks at 2θ = 34.8°, 61.3° and 68.5° were ascribed to Ti_5_Si_3_. In addition, the peaks at 2θ angles of 37.1° and 73.5° correspond to the Ti_3_SiC_2_ crystal planes of (103) and (1012). For S1200, the characteristic peaks appearing at 2θ angles of 37.1°, 39.7°, 40.9°, 42.7°, 54.2°, 60.5° and 76.9° matched very well with the Ti_3_SiC_2_ crystal planes of (103), (104), (008), (105), (108), (110) and (205), according to the standard card of JCPDS No.48-1826, indicating that this ternary compound has been successfully obtained in the final product.

**Figure 2:**
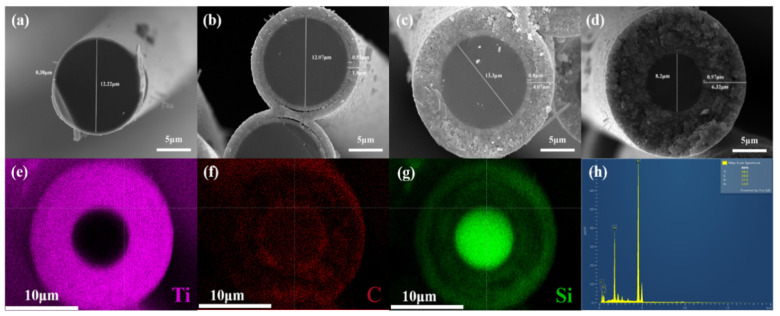
Cross-sectional morphologies of SiC fibers: (**a**) S900; (**b**) S1000; (**c**) S1100; (**d**) S1200; (**e**–**g**) EDX (Energy Dispersive X-Ray Spectroscopy) elemental mapping of S1200; (**h**) map sum spectrum of S1200.

Figure 2 shows the cross-section morphologies of TiC-Ti_3_SiC_2_ bilayered coating silicon carbide fibers. The images clearly showed that the coatings were homogeneous in thickness and adherent to the fibers along the length, as also along the diameter of the fibers. As can be seen in the Figure 2, the surfaces of all samples were smooth and showed no cracks. In addition, the coating layers were divided into two layers, except for S900. The thicknesses of coating layers for S900 to S1200 were gradually increased, reaching 0.38, 1.8, 4.07 and 6.32 μm, respectively. The thicknesses of the inner layer were increased from 0.55 μm for S1000 to 0.97 μm for S1200, while the thicknesses of the outer layer for S1000, S1100 and S1200 showed the same trend, with 1.25 μm, 3.27 μm and 5.45 μm, respectively. Noticeably, the diameter of SiC fiber decreased significantly after annealing at 1200 °C, from 12.22 μm for S900 to 8.2 μm for S1200. To explain this phenomenon, the cross-section of S1200 was analyzed by energy spectrum analysis, as can be seen in Figure 2e–h. The fiber was composed of 39.2 at% Ti, 30.9 at% C,17.3 at% O and 12.6 at% Si, indicating that Ti was successfully introduced into SiC fiber. In addition, Ti elements were evenly distributed on the interface without any change in composition gradient along with the fiber radially, but the contents of C and Si elements had obvious changes along the fiber diameter. The 0.97 μm thick inner layer of S1200 was rich in C and lacking in Si, indicating the existence of TiC. Then, the C content began to decrease and the Si content increased slightly, which represented the emergence of Ti_3_SiC_2_. The outermost 3 μm saw a sharp increase in Si content. Decomposition of Ti_3_SiC_2_ of S1200 is thought to occur by the following reaction: Ti_3_SiC_2_ → 2TiC + Ti + Si.

### 3.2. Dielectric Loss Properties

To further explore the influence of the introduction of TiC-Ti_3_SiC_2_ bilayered coating on the microwave absorbing performance of the SiC fibers, the complex permittivity and permeability of SiC fibers within a frequency of 2–18 GHz were evaluated. Because all the samples are non-magnetic, the magnetic responses are so weak that they could be neglected. Therefore, dielectric loss plays a dominant role in microwave absorbing properties.

**Figure 3:**
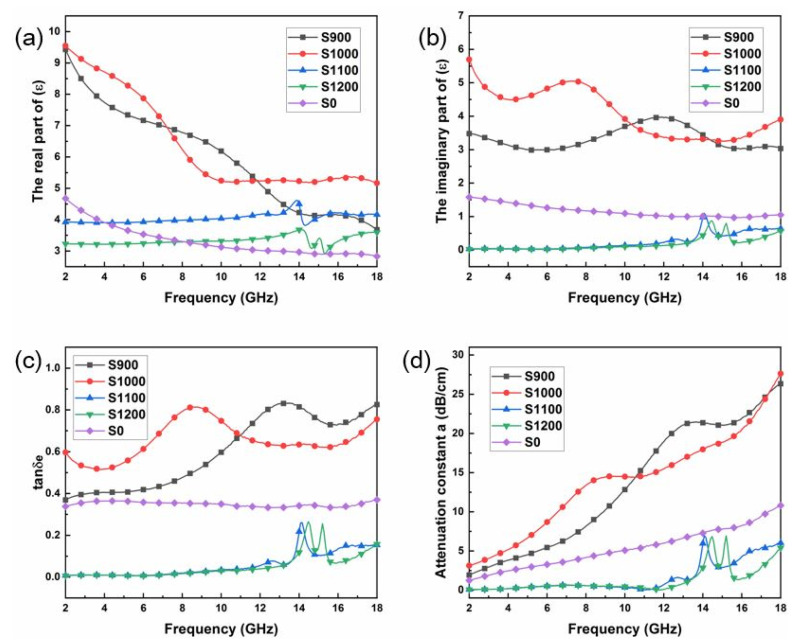
Dielectric loss properties of SiC fibers: (**a**) the real part of relative complex permittivity; (**b**) the imaginary part of relative complex permittivity; (**c**) tangent loss of (*ε*″/*ε*′); (**d**) attenuation constant.

Figure 3a,b display the frequency dependence of the real (*ε*′) and imaginary (*ε*″) parts of the relative complex permittivity of the SiC fibers, respectively. For the pristine SiC in the tested region, with the increase in frequency, the *ε*′ value decreased from 4.8 to 2.8, while the *ε*″ value fluctuates around 1. The *ε*′ values of the S900 and S1000 were found to be significantly improved compared to S0, while the *ε*′ values of S1100 and S1200 were lower than that of S0. The enhancement of permittivity of the SiC fibers indicates the increased ability of electric energy storage. Generally, higher *ε*″ values are expected for absorbers. The *ε*″ value plots of the samples from S0 to S1200 are displayed in Figure 3b. The *ε*″ values of S1000 were the highest among those of the samples with the *ε*″ value of 9.8. Another important feature that should be noticed is that an obvious frequency dispersion phenomenon (or in other words, obvious fluctuation of the dielectric spectrum at specific frequencies) could be observed, especially for the samples of S900 and S1100. Obvious frequency dispersion is favorable for microwave attenuation. Owing to the good electrical conductivity of TiC, many conductivity paths for electrons can be formed at the surface of fibers. In addition, the TiC layer means more defects in the interface structure which results in generating sufficient interface polarization and increased dipole polarization in the fibers. Both of these factors contribute to the increased permittivity of SiC fibers. Figure 3c displays the plots of the tanδe (tanδe = *ε*″/*ε*′) vs. frequency for all samples. The tanδe of S900 and S1000 are higher than that of S0, corresponding to the superior electromagnetic wave attention capability. Figure 3d shows the attenuation constants of electromagnetic waves in the SiC fibers, which is obtained through the following equation [28]:

(1) $$a=\frac{\sqrt{2}\pi f}{c}\sqrt{\left(\mu^{\prime \prime }\varepsilon^{\prime \prime }-\mu^{\prime }\varepsilon^{\prime }\right)+\sqrt{{\left(\mu^{\prime \prime }\varepsilon^{\prime \prime }-\mu^{\prime }\varepsilon^{\prime }\right)}^{2}+{\left(\mu^{\prime }\varepsilon^{\prime \prime }+\mu^{\prime \prime }\varepsilon^{\prime }\right)}^{2}}}$$

The results are consistent with Figure 3c. The samples of S900 and S1100 show quite strong dielectric loss properties, among others.

### 3.3. Specular Reflection Suppression

The specular reflection suppression characteristics of a microwave absorbing sheet has been well described by reflection loss (*RL*). For a single layer of RAM attached on a metal plate, the *RL* can be obtained based on its complex permittivity and permeability through transmission line theory. For a normal incident case, the *RL* is independent of polarization, and it is calculated by the following equations [29,30,31]:

(2) $$RL=20\log\left|\frac{Z_{in}-Z_{0}}{Z_{in}+Z_{0}}\right|$$

where $Z_{0}$ is the impedance of free space and $Z_{in}$ is the input impedance at air–material interface when the microwave absorbing sheet is terminated by the metal plate. The input impedance for a normal incident case can be expressed as:

(3) $$Z_{in}=Z_{0}\sqrt{\mu_{r}/\varepsilon_{r}}\tanh\left[j\left(2\pi f/c\right)\sqrt{\mu_{r}\varepsilon_{r}}d\right]$$

where *c* is the velocity of light, *d* is the thickness of the absorbing material, *f* is the electromagnetic wave frequency, and $\varepsilon_{r}$ and $\mu_{r}$ are complex relative permittivity and permeability, respectively.

In fact, the electromagnetic parameters and thickness of the absorbing material and microwave frequency simultaneously affect the input impedance. The impedance matching characteristic parameter can well clarify the mechanism and is calculated by the following equation [32]:

(4) $$\left|\frac{Z_{in}}{Z_{0}}\right|=\left|\sqrt{\frac{\mu_{r}}{\varepsilon_{r}}}\tanh\left[j\left(2\pi f/c\right)\sqrt{\mu_{r}\varepsilon_{r}}d\right]\right|$$

In general, the minimum *RL* can be obtained when the corresponding $\left|Z_{in}/Z_{0}\right|$ is equal or close to 1.

Apart from the constitutive electromagnetic properties of RAMs, the thickness can greatly influence *RL* due to the theory of quarter-wavelength cancellation. The relationship between the matching thickness $t_{m}$ and matching frequency $f_{m}$ under the quarter-wavelength cancellation condition is [29]:

(5) $$t_{m}=nc/\left(4f_{m}\sqrt{\mu_{r}\varepsilon_{r}}\right)\left(n=1,3,5\ldots \right)$$

When the thickness satisfies Equation (5), two emerging reflected waves from the air–material interface and material–metal interface are out of phase by 180°, leading to an extinction of them at the air–material interface.

**Figure 4:**
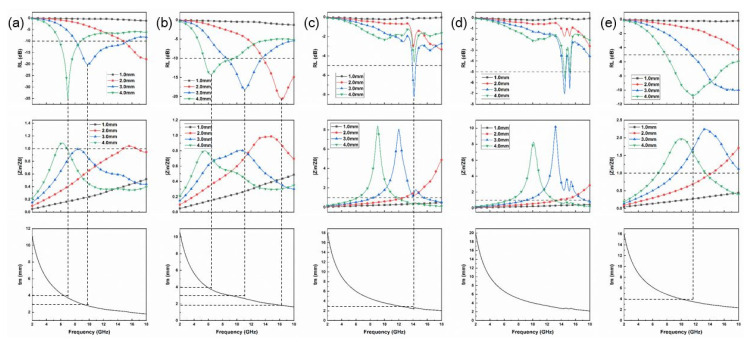
The specular reflection suppression of SiC fibers (reflection loss (picture in first line), impedance matching characteristics (picture in second line), the quarter-wavelength condition (picture in third line)): (**a**) S900, (**b**) S1000, (**c**) S1100, (**d**) S1200, (**e**) S0.

Figure 4 shows the specular reflection suppression of the SiC fiber samples, which can be obtained from Equation (2) to (5). As shown in Figure 4e, the microwave absorption of the pure SiC fiber has poor absorption performance, the minimum reflection loss (*RL*_min_) exhibits unsatisfactory values as low as −11 dB at 12 GHz, and even the thickness reaches 4 mm. Notably, incorporation with TiC-Ti_3_SiC_2_ bilayered coating on the surface of SiC fibers could promote specular reflection suppression performance greatly in terms of both the EABW (effective absorption bandwidth, *RL <* −10 dB) and EM absorption intensity. As shown in Figure 4a,b, evidently, the EABWs were 7.36 GHz and 5.04 GHz for S900 and S1000 samples with the matched thickness of 3 mm, respectively. Especially, the optimal *RL*_min_ value of S900 is as strong as −34.75 dB at 6.96 GHz with a thickness of 4 mm. However, a further increase of heat-treatment temperature does not bring further enhanced specular reflection suppression, which is illustrated in Figure 4c,d. This is mainly attributed to fact that higher heat-treatment temperature, corresponding to a higher volume fraction of the metal Ti_3_SiC_2_ component, leads to more direct reflection of the incident wave rather than absorption.

On the other hand, the S900 composites possess the best impedance matching of all the samples. For instance, the $\left|Z_{in}/Z_{0}\right|$ values between 0.8 and 1 for all composites covered a broader frequency band at different thicknesses. Thus, the S900 samples should achieve a strong absorption peak. This result was in good agreement with the achieved *RL* values. Combined with the analytical results of measured electromagnetic parameters and *RL* values, it can be concluded that the form of TiC to the SiC can appropriately regulate the permittivity, consequently enhancing electromagnetic attenuation capability, optimizing the impedance matching and thereby finally improving the specular reflection suppression performance.

Favorable specular reflection suppression performances including the broadband EABW, the stronger absorption intensity, and the relatively thin matched thickness make the TiC-Ti_3_SiC_2_@SiC composites promising microwave absorbers, which means greater potential for practical application in the field of military radar stealth and civil electromagnetic compatibility.

### 3.4. Non-Specular Scattering Suppression

The non-specular scattering suppression characteristics of absorbing material are characterized by surface wave attenuation constants. Different from specular reflection suppression, surface waves are attenuated along the tangential direction of the absorbing layer. For a single layer of RAM attached on a metal plate, the surface wave attenuation constants of TM type can be obtained through solving the following dispersion equations, in which complex transcendental relations exist [33,34]:

(6) $$D\left(k_{0},\beta \right)\equiv \sqrt{k_{0}^{2}\varepsilon_{r}\mu_{r}-\beta^{2}}\tan\left(d\sqrt{k_{0}^{2}\varepsilon_{r}\mu_{r}-\beta^{2}}\right)+j\varepsilon_{r}\sqrt{k_{0}^{2}-\beta^{2}}=0$$

In the above dispersion equations, $k_{0}$ is the wave number of free space, and is given by:

(7) $$k_{0}=2\pi f\sqrt{\varepsilon_{0}\mu_{0}}$$

where $\varepsilon_{0}$ and $\mu_{0}$ are the permittivity and permeability in air, respectively. The quantity β is the longitudinal wave number or the propagation constant of the surface wave. The real part $\left(\beta^{\prime }\right)$ and imaginary part $\left(\beta^{\prime \prime }\right)$ of β are the phase constant and attenuation constant of the surface wave, respectively. Through the Helmholtz wave equation, these wave numbers are connected by the following relations:

(8) $$k_{1}^{2}+\beta^{2}=k_{0}^{2}$$

(9) $$k_{2}^{2}+\beta^{2}=k_{0}^{2}\varepsilon_{r}\mu_{r}$$

The quantities $k_{1}$ and $k_{2}$ are the transverse wave numbers of the surface wave field outside and inside the absorbing layer, respectively. Then, the quantity β can be obtained through solving the dispersion in Equation (6) by iterative procedure, and then $k_{1}$ and $k_{2}$ can be calculated by solving (8) and (9).

**Figure 5:**
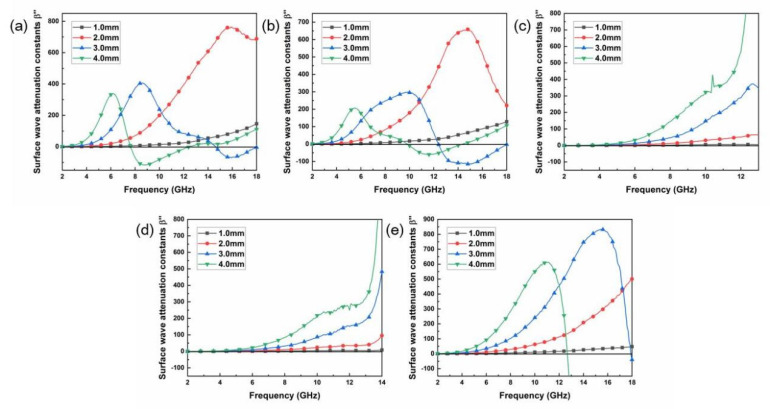
The non-specular scattering suppression of SiC fibers (surface wave attenuation constants β”): (**a**) S900, (**b**) S1000, (**c**) S1100, (**d**) S1200, (**e**) S0.

The surface wave attenuation constants of the SiC fiber samples are shown in Figure 5a–e. As shown in Figure 5a,b, it is found that the surface wave attenuation constants first increase to it maxima, then decrease to zero and finally become negative values. The frequency when the surface wave attenuation constants become zero is called the upper cutoff frequency. The negative values mean that the electromagnetic energy is amplified rather than absorbed, and therefore must be ignored. Negative values result from the non-physical solution of the dispersion equation, which mean that surface waves cannot be excited and thus no propagation occurs along the layer [33]. It is observed that all 1.0 mm-thick samples show relatively weak surface attenuation ability within 2–18 GHz. As the layer thickness increases, the attenuation curve gradually presents as a narrower peak since the cutoff frequency shifts to a lower frequency with the increase of thickness. For S900 and S1000 samples, it can be applied to surface wave attenuation when the thickness is 2.0 mm, while the thickness needs to be increased to 3.0 mm for S1100 and S1200 samples.

## 4. Conclusions

In this work, TiC-Ti_3_SiC_2_ bilayered coatings were successfully grown on the surfaces of SiC fibers by an in situ molten salt process. The *ε*′ values of the S900 and S1000 were found to be significantly improved compared to S0, while the *ε*′ values of S1100 and S1200 were lower than that of S0. The electromagnetic loss properties show the same trend, the tangent loss of *ε*″/*ε*′ of S900 and S1000 were greater than S0, while S1000 and S1200 were smaller. The specular reflection suppression for the SiC fibers with TiC-Ti_3_SiC_2_ bilayered coating is greatly enhanced in terms of both the EABW and microwave absorption intensity for S900 and S1000. Meanwhile, the non-specular scattering suppression properties were also evaluated, which is in agreement with the specular reflection suppression performance. The enhanced microwave absorbing performance of the Ti_3_SiC_2_ coated SiC fibers is mainly attributed to the improved impedance matching, as well as dissipation resulting from hopping migration. This finding demonstrates that the Ti_3_SiC_2_ coated SiC fibers are considered to be a promising candidate for a novel solution with favorable thickness and a light weight in radar stealth in the future.
